# Hyperglycemia exacerbates colon cancer malignancy through hexosamine biosynthetic pathway

**DOI:** 10.1038/oncsis.2017.2

**Published:** 2017-03-20

**Authors:** A Vasconcelos-dos-Santos, H F B R Loponte, N R Mantuano, I A Oliveira, I F de Paula, L K Teixeira, J C M de-Freitas-Junior, K C Gondim, N Heise, R Mohana-Borges, J A Morgado-Díaz, W B Dias, A R Todeschini

**Affiliations:** 1 Instituto de Biofísica Carlos Chagas Filho, Rio de Janeiro, Brazil; 2 Instituto de Bioquímica Médica Leopoldo de Meis, Universidade Federal do Rio de Janeiro, Rio de Janeiro, Brazil; 3 Programa de Biologia Celular, Instituto Nacional de Câncer (INCA), Rio de Janeiro, Brazil

## Abstract

Hyperglycemia is a common feature of diabetes mellitus, considered as a risk factor for cancer. However, its direct effects in cancer cell behavior are relatively unexplored. Herein we show that high glucose concentration induces aberrant glycosylation, increased cell proliferation, invasion and tumor progression of colon cancer. By modulating the activity of the rate-limiting enzyme, glutamine-fructose-6-phosphate amidotransferase (GFAT), we demonstrate that hexosamine biosynthetic pathway (HBP) is involved in those processes. Biopsies from patients with colon carcinoma show increased levels of GFAT and consequently aberrant glycans’ expression suggesting an increase of HBP flow in human colon cancer. All together, our results open the possibility that HBP links hyperglycemia, aberrant glycosylation and tumor malignancy, and suggest this pathway as a potential therapeutic target for colorectal cancer.

## Introduction

Colorectal cancer (CRC) is the third most common cancer and the second leading cause of cancer death in the United States.^1^ Epidemiological evidences show that individuals with diabetes mellitus (DM) have significantly higher risk of developing multiple types of cancers.^2, 3, 4, 5^ Furthermore, DM correlates tightly with the incidence and mortality of CRC.^6, 7, 8, 9^

Hyperglycemia is the most important feature of DM, a hallmark for both DM1 and DM2. The excess glucose supports cancer cells’ increased energetic and biosynthetic needs.^10^ It has been reported that high glucose (HG) triggers several direct and indirect mechanisms that cooperate to promote cancer progression, such as induction of epithelial mesenchymal transition (EMT),^11^ increased levels of insulin/IGF-1 and inflammatory cytokines in circulation,^12, 13, 14^ increased leptin and pro-survival AKT/mTOR signaling^15^ and enhancement of WNT/β-catenin signaling.^16^ Together, these studies reveal that hyperglycemia *per se* has an important impact on cancer cells.

Most malignant tissues have increased fludeoxyglucose uptake associated with an increased rate of glycolysis and glucose transportation.^17, 18^ This high glycolytic rate likely benefits proliferating cells through the production of glycolytic intermediates, which fuels metabolic pathways that generate *de novo* biosynthesis of nucleotides, NADPH, lipids, amino acids and glycoconjugates. Despite the fact that the majority of glucose enters glycolysis, ~2–5% of a cell's glucose enters the hexosamine biosynthetic pathway (HBP), which provides UDP-*N*-acetyl-D-glucosamine (UDP-GlcNAc), and its derivative UDP-*N*-acetyl-D-galactosamine (UDP-GalNAc), and CMP-*N-*acetylneuraminic acid (CMP-Neu5Ac).^19^ These compounds are substrates for *N*- and *O*-glycosylation of extracellular proteins and glycolipids, and for the post-translational modification of intracellular proteins by *O*-GlcNAc transferase (OGT). UDP-GlcNAc is highly responsive to variations in cell nutrients as its synthesis depends on products of the metabolism of glucose, amino acids, fatty acids and nucleotides.^20^ Hence, UDP-GlcNAc can serve as a reporter for the functional status of multiple pathways, and is considered an ideal metabolic sensor.^21^

The rate-limiting enzyme in glucose flux through the hexosamine pathway is glutamine-fructose-6-phosphate amidotransferase (GFAT), which catalyzes the synthesis of glucosamine-6-phosphate from fructose 6-phosphate and glutamine. GFAT has two isoforms in mammals, GFAT1 and GFAT2, which are expressed in different tissues and possibly have different functions.^22^ Studies have shown a growing association of GFAT with metabolic diseases such as DM^23, 24^ and cancer.^11, 20^ In a previous study, we demonstrated that increasing the flow of metabolites through HBP, by increasing extracellular glucose concentrations or overexpressing GFAT2, provokes aberrant glycosylation of fibronectin and induces EMT in human alveolar epithelial adenocarcinoma cells (A549).^11^ Recently, we demonstrated that glucose is shunted to HBP during the EMT changing the glycophenotype of A549 cells.^20^ Together, our results indicate that cell glycosylation senses glucose changes in the microenvironment and modulates cell plasticity.

Glycoconjugates have critical roles in different cellular processes including cellular adhesion, migration, growth, differentiation, signal transduction, receptor activation, immune response modulation, and are targets of antibodies used in cancer diagnosis and therapy.^21, 25^ Growing evidence shows that aberrant glycosylation confers adaptive advantage to cells during oncogenic transformation in CRC.^26, 27^ However, the direct effects of hyperglycemia in cancer cell behavior are relatively unexplored. Our hypothesis is that HG increases colon cancer malignancy by altering cell glycosylation through HBP. Here, we show that colon adenocarcinoma cells in HG presented aberrant glycosylation and increased proliferation, invasion and tumor growth. In addition, hyperglycemic mice showed an exacerbated tumor growth and metastasis. Modulation of GFAT activity leads to significant reduction of tumor growth, cell motility and metastasis. Furthermore, we showed higher levels of GFAT in human colon cancer samples compared to normal adjacent tissues. The results shown here suggest an important role of HBP for tumor progression of colon cancer, and uncover a possible new target for cancer chemotherapy.

## Results

### High glucose concentration increases cell growth and invasion

**Figure 1:**
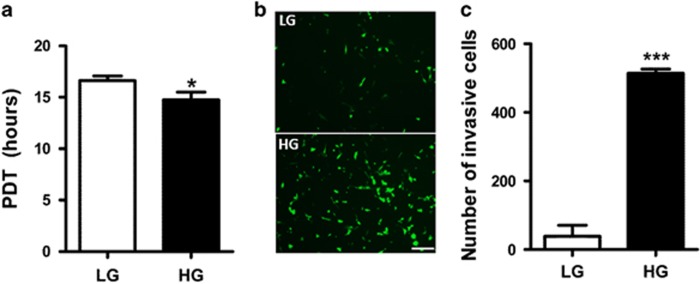
Hyperglycemia increases proliferation and invasion in MC38 cells. (**a**) Population doubling time (PDT) of MC38 cells cultured in high (HG) or low glucose (LG) concentration. *n*=(3); error bars indicate mean±se.m. (**b**) Photomicrography of lower chamber of transwell Matrigel-coated membrane of (LG and HG) MC38-GFP cells. (**c**) All cells that invaded to the lower chamber were counted after 24 h of incubation. *n*=3; ***P<0.0001 *t* test; scale bar, 100 μm.

To test the effects of HG on cancer progression, colon adenocarcinoma MC38 cells were cultured in media containing low glucose (LG; 5 mM) or HG (25 mM) concentration for over 30 days. MC38 cells cultured in HG (MC38-HG) presented increased proliferation when compared with cells cultured in LG (MC38-LG) as analyzed by population doubling time (PDT; Figure 1a). We found that HG induced the invasive potential of MC38-GFP^+^cells in transwell membranes coated with matrigel when compared to LG condition (Figures 1b and c).

### Hyperglycemia governs tumor growth, homing and metastasis *in vivo*

**Figure 2:**
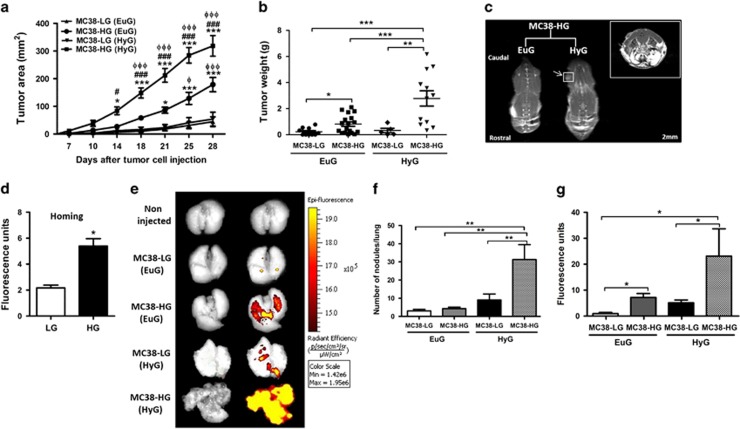
Hyperglycemia exacerbates tumor progression *in vivo*. (**a**) Tumor area and (**b**) weight of MC38-LG or MC38-HG injected in the flank of euglycemic (EuG) and hyperglycemic (HyG) mice. *n*=9–18 per group; error bars indicate mean±s.e.m.; *P<0.05, **P<0.001, ***P<0.0001, ^#^*P*<0.05, ^###^*P*<0.0001; (*) represents comparison between MC38-HG and MC38-HG (HyG) with MC38-LG; (#) is relative to comparison between MC38-HG and MC38-HG (HyG). Anova two way, Bonferonni post test. (**c**) Magnetic resonance whole body of euglycemic (EuG) and hyperglycemic (HyG) mice, 5 days after subcutaneous MC38-HG cells injection. (**d**) Fluorescence levels of lung of euglycemic mice, 3 days after injection of MC38-GFP-LG or MC38-GFP-HG cells into lateral tail vein. *n*=3 per group. (**e**) Representative fluorescent images of lung 21 days after MC38-GFP cell infusion. The picture shows five distinct groups: Non-injected mice; euglycemic mice (EuG) injected with MC38-LG or MC38-HG cells; hyperglycemic mice (HyG) injected with MC38-HG or MC38-LG. After the acquisition of images, the nodules were counted (**f**) and lung was homogenized and diluted for a fluorescence read-out (**g**); *n*=5 per group, *P<0.05; **P<0.001;±s.e.m.; one-way ANOVA; Tukey's multiple comparison test.

We next explored the MC38-LG or MC38-HG cells’ potential to induce tumor growth through ectopic injection in the flank of Euglycemic C57BL/6 mice (EuG). Results demonstrated a larger growth area and weight in the mice injected with MC38-HG cells when compared to mice injected with LG cells (Figures 2a and b).

Hyperglycemia was induced *in vivo* by selective destruction of β-pancreatic cells with streptozotocin (STZ) treatment of C57BL/6 mice. Hyperglycemic mice displayed blood glucose levels threefold greater than those of the control animals (*P*<0.0001) (Supplementary Figure S1A). MC38-LG cells injected in hyperglycemic mice (HyG) do not present tumor growth potential. However, the HyG mice injected with MC38-HG cells present a tumor growth exacerbated when compared to other groups (Figures 2a and b). HyG mice tumors (Figures 2a and b) occurred earlier in 60% of the cases as shown by whole-body magnetic resonance (11 of 18; Figure 2c).

Injecting MC38-GFP^+^ cells into lateral tail vein of EuG or HyG mice, we assessed the impact of hyperglycemia in cell homing and metastasis. First, we observed an increase of MC38-HG GFP^+^ cells’ homing to lung 3 days after injection into EuG mice compared to that of MC38-LG GFP^+^ cells (Figure 2d). The metastasis was analyzed 21 days after cell injection. Figures 2e and f show the images of the lung and number of metastatic nodules of the different groups. Next, the organs were homogenized and diluted for fluorescence read-out quantification (Figure 2g). Lungs of HyG mice were almost completely overtaken by metastatic nodules of MC38-HG injected cells, and correspondingly high fluorescence measurements were obtained. Besides, incubation of MC38 cells cultured in HG medium was sufficient to induce metastasis in the lungs of EuG mice. Furthermore, metastatic spread of MC38-HG cells was statistically significant as EuG mice that received MC38-LG cells presented fewer metastatic foci and lower fluorescence detection. High blood glucose contributed to increase in cell metastasis as HyG mice presented more nodules than EuG mice injected with MC38-LG cells did. These results suggest that HG induces phenotypic alterations on MC38 cells that regulate cell proliferation, homing and metastasis.

### High glucose induces aberrant glycosylation

**Figure 3:**
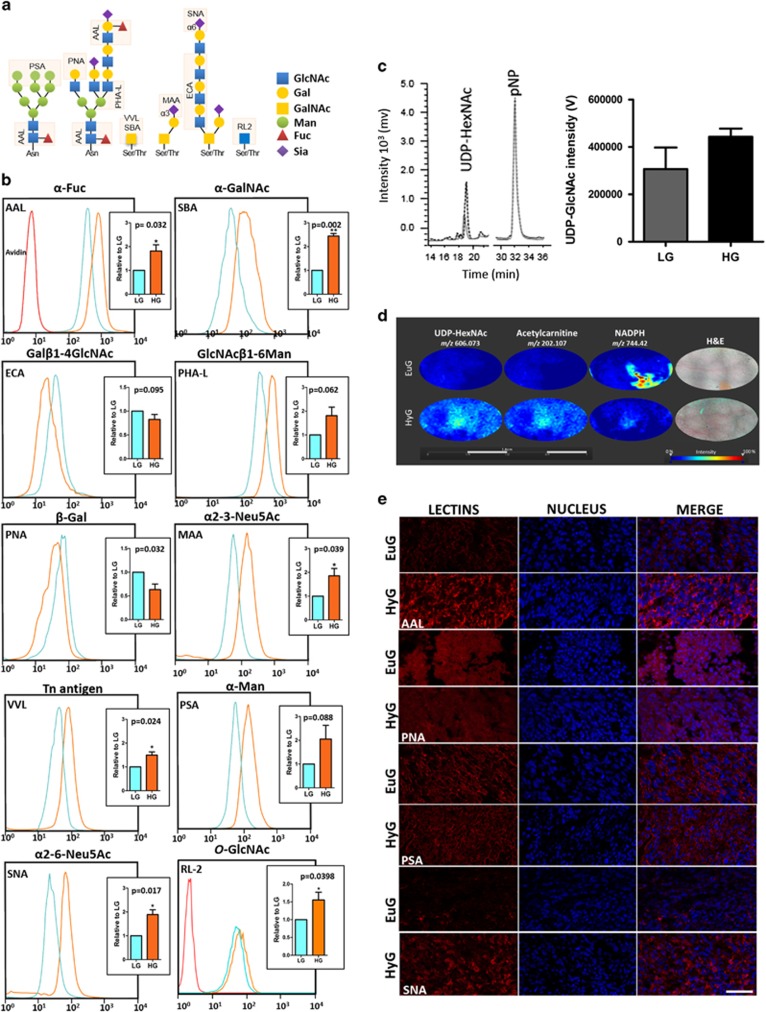
Hyperglycemia induces aberrant glycosylation. (**a**) Scheme representing binding specificities of the lectins and RL2 antibody used in this study. (**b**) Flow cytometry histograms show representative binding profile of different lectins in MC38 cells cultured in high (HG, orange) or low glucose (LG, blue) concentration. Red empty histogram refers to cells stained with the FITC-conjugated streptavidin, and bar graph shows the differences between the fluorescence intensity for each lectin. *O*-GlcNAc labeling was accessed by immunolabeling with RL2 antibody, and its red histogram represents cells stained with Alexa Fluor 488-conjugated antibody. The results represent four experimental replicates. (*n*=4); unpaired *t*-test. (**c**) Left, chromatograms of polar metabolites of cell extracts from MC38-LG (gray line) and MC38-HG cells (black dashed line), showing regions corresponding to UDP-HexNAc and pNP retention times. Right, UDP-HexNAc quantification of MC38-LG (gray) and MC38-HG cells (black). Quantitative analyses are shown as mean±s.d. of two independent experiments. (**d**) Comparison of *m/z* localization in subcutaneous tumor from euglycemic and hyperglycemic mice. MALDI-MSI analysis showing the distribution of *m/z* 606.073 (UDP-hexosamine), *m/z* 202.10 (acetylcarnitine) and *m/z* 743.074 (NADPH) and right panels are H&E-stained. (**e**) Lectin binding to tumor tissue of hyperglycemic versus euglycemic mice. Photomicrografies are representative of subcutaneous tumor tissue from hyperglycemic (HyG) versus euglycemic (EuG) mice analyzed 28 days after MC38 implantation. Scale bar, 100 μm. (*n*=3).

Alterations in cellular glycosylation confer advantages in terms of tumor growth, tumor dissemination and immune escape.^28^ Evidence demonstrated that hyperglycemia induces biosynthesis of aberrant glycoconjugates.^11, 21^ Therefore, we investigated the glycophenotype changes of MC38 cells cultured in LG or HG by flow cytometry using an array of sugar-binding proteins (lectins, Supplementary Table SI) that identify alterations on cell surface glycosylation seen during tumorigenesis (Figure 3a). Effect of hyperglycemia on biosynthesis of glycoconjugates was time dependent (Supplementary Figure S2), and Figure 3b shows a significant increase of *N*-glycans wherein the α-mannose (α-Man) of the saccharide core was branched with β1-6-GlcNAc, generating triantennary *N*-glycans (Phytohemagglutinin-L, PHA-L; *P*=0.0615). We also observed an increase of un-substituted α-GalNAc units, *O*-linked to the core protein, known as Tn antigen (*Vicia villosa* Lectin, VVL; *P*=0.0243). This observation is in agreement with the increased binding of Soybean agglutinin to α-GalNAc residues (*P*=0.0018). Moreover, HG concentration induced cell surface hypersialylation as seen by the increase of glycoconjugates terminated with α2-6- and α2-3-linked Neu5Ac, implied by staining with *Sambucus nigra* agglutinin (SNA; *P*=0.0170), and *Maackia amurensis* agglutinin (MAA; *P*=0.0170) respectively. Increase of sialic acid expression was accompanied with reduction of terminal β-galactopyranose (β-Gal*p*) unities recognized by the Peanut agglutinin (PNA; *P*=0.0324). In spite of increase of saccharide unities derivate from hexosamine pathway, augmented expression of glycoconjugates containing α-fucose (α-Fuc) observed by the increase of *Aleuria aurantia* agglutinin (AAL; *P*=0.0329) binding was also detected (Figure 3b). Hyperglycemia induces the same glycophenotypic changes in the 4T1 murine mammary carcinoma cell line from a BALB/cfC3H (Supplementary Figure S3) revealing that HG effect on glycosylation is not cell specific.

Results showing that HG increases the biosynthesis of oligosaccharides carrying hexosamines suggest an increase in HBP flux. As the levels of GFAT1 or 2 do not increase under HG concentrations (Supplementary Figure S4), we analyzed the impact of hyperglycemia in the biosynthesis of the final product of HBP, UDP-GlcNAc. Using liquid chromatography with porous graphitic column, we were able to demonstrate that cells cultured in HG *in vitro* synthesize more UDP-hexosamines (UDP-GlcNAc and UDP-GalNAc) than MC38 cells cultured in LG (Figure 3c). By using high-resolution matrix-assisted laser desorption/ionization Fourier-transform ion cyclotron resonance mass spectrometry imaging (MALDI-FT-ICR MSI) on tissue samples, we accessed the impact of hyperglycemia in production of UDP-GlcNAc in subcutaneous tumors from EuG and HyG mice. Matrix-assisted laser desorption/ionization mass spectrometry imaging (MALDI-MSI) is a powerful technique combining mass spectrometry with histology, allowing for the spatially resolved and label-free detection of hundreds to thousands of compounds within a single tissue section.^29^ Figure 3d shows localizations for different *m/z* values in subcutaneous tumors from EuG and HyG mice. From Figure 2c it is clear that the [M−H]^−^ *m/z* 606.073 relative to UDP-hexosamines is mostly present on tissue from HyG mice. In contrast, NADPH (*m/z* 743.074), a product of pentose phosphate pathway, is present across both samples, but most intensely in tumor from EuG mice. It is noteworthy that the *m/z* 606.073 presents a distribution similar to that of the ion *m/z* 202.107 related to acetylcarnitine, a marker for hypoxic tumor regions.^30^ Therefore, sustained hyperglycemia increases UDP-GlcNAc biosynthesis. In agreement with alterations in the pool of activated hexosamines, histochemistry of subcutaneous tumors showed an increase of glycoconjugates containing α2-6-linked Neu5Ac residues (SNA), with simultaneous reduction of PNA binding to terminal β-Gal*p* unities (Figure 3d). Besides, increase of α-Fuc residues (AAL) corroborates with *in vitro* results.

### GFAT determines tumor growth, invasion and aberrant glycosylation

**Figure 4:**
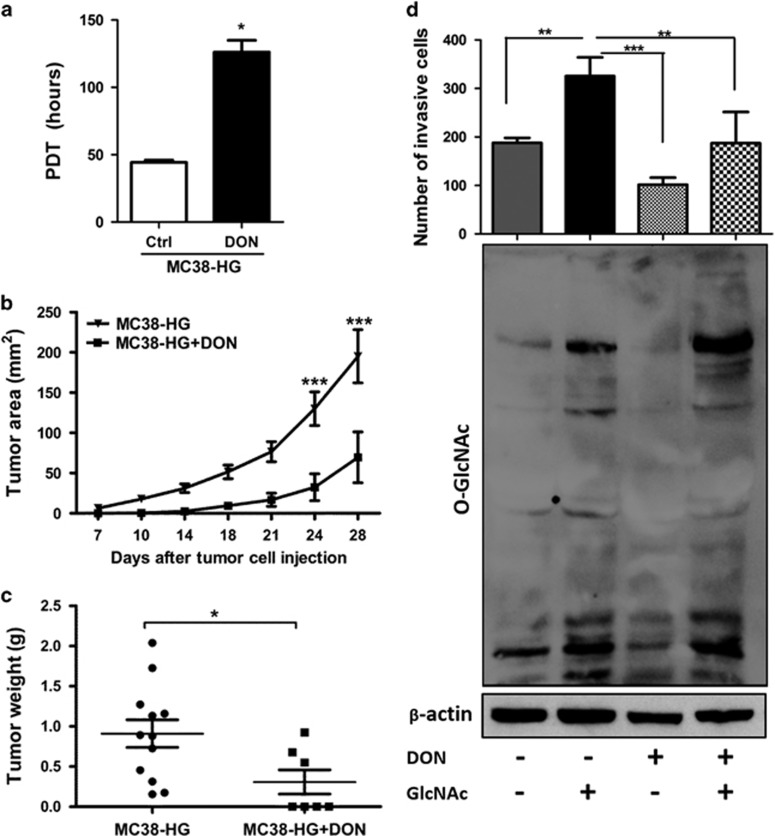
Pharmacological inhibition of GFAT decreases tumor growth and invasion. (**a**) Proliferation analysis of MC38-HG cells cultured in the absence (Ctrl) or presence of 1 μM DON (DON). (*n*=3); error bars indicate mean±s.e.m.; *t*-test. (**b**) Tumor area and (**c**) weight of MC38-HG cells untreated, or treated, with 1 μM DON (MC38-HG+DON) and injected in the flank of euglycemic mice. MC38-HG (*n*=12); MC38-HG+DON (*n*=7). (**d**) *Lower panel*. Western blot analysis of lysate from MC38-LG cells treated with Mannitol (osmotic control), GlcNAc, DON and DON+GlcNAc analyzing *O*-GlcNAc levels and β-actin expression. *Upper panel*. Invasion of MC38-LG cells treated with Mannitol (osmotic control, 40 mM), GlcNAc (40 mM), DON (1 μM) and DON+GlcNAc by transwell analysis of 24 h of incubation; *n*=3,±s.e.m.; one-way ANOVA, Bonferroni's multiple comparison test *P<0.05; **P<0.001; ***P<0.001

To gain insight into whether effect of HG on tumor progression is associated to glucose assimilation into HBP, we pre-treated the GFP-MC38-HG cells with the pharmacologic inhibitor of GFAT, 6-Diazo-5-oxo-L-norleucine (DON). DON treatment decreased MC38-HG proliferation, as measured by PDT assay (Figure 4a). DON significantly impaired tumor growth of MC38-HG treated cells injected in the mice flank of EuG mice (Figures 4b and c). Moreover, DON presented a tendency to decrease the homing to lung 3 days after cell injection (Supplementary Figure S5). Besides, DON treatment reduced the invasiveness of MC38 cells in transwell membranes coated with matrigel (Figure 4d, upper panel). As DON is not a specific inhibitor of GFAT, as it inhibits other amidotransferases, we tested whether the addition of GlcNAc can restore cell invasion by bypassing GFAT inhibition by DON. It is well established that *O*-GlcNAc is highly responsive to the HBP.^31^ Our results clearly showed that DON decreased *O*-GlcNAc levels, whereas the addition of GlcNAc can increase the *O*-GlcNAc levels, even in the presence of DON (Figure 4d, lower panel), indicating that the DON effect observed in our model is due to HBP inhibition. As illustrated in Figure 4d (upper panel), GlcNAc increases cell invasion; besides, addition of GlcNAc to DON-treated MC38-HG cells partially restores the ability of cells to invade, thus, decreasing DON inhibition. These results reinforce the role of GFAT in tumor progression.

**Figure 5:**
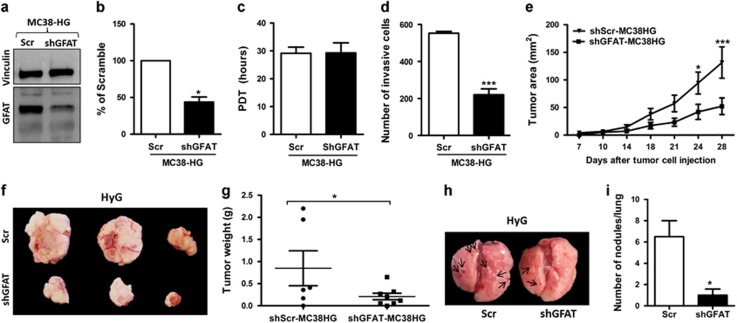
Effect of GFAT deletion on tumor progression. (**a**, **b**) Protein expression of GFAT in shGFAT-MC38 cells. (*n*=4). (**c**) The population doubling time (PDT) analysis of Scrambled MC38-HG cells related to shGFAT MC38-HG cells. (**d**) The Matrigel-coated chamber transwell invasion assay of shGFAT MC38-HG cells compared to that of Scrambled MC38-HG cells. (**e**) Tumor area, (**f**) representative images and (**g**) weight of euglycemic (EuG) mice injected subcutaneously with shGFAT MC38-HG cells or Scrambled MC38-HG cells (*n*=6–8). (**h**) Representative images and (**i**) quantification of lung metastatic nodules in hyperglycemic (HyG) mice injected with shGFAT MC38-HG cells or scrambled MC38-HG cells (*n*=5). Results are expressed as mean±s.e.m.; two-tailed, unpaired *t*-test and Anova two way; Bonferonni post test. *P<0.05, ***P<0.001.

To further substantiate the role of GFAT in tumor progression induced by hyperglycemia, we assessed the impact of its gene knockdown in the MC38-HG cells (Figures 5a and b). In agreement with above results using DON, downregulation of GFAT protein levels reduced tumor growth *in vivo* (Figures 5e–g), although it appears to have no effect in the proliferation assay (Figure 5c). The high invasion capacity observed in the HG cells (Figures 1b and c) was strongly decreased in the shGFAT group (Figure 5d). Moreover, the experimental metastasis analysis shows that GFAT deficiency significantly attenuates metastatic spread of shGFAT-MC38 cells to the lungs of HyG animals compared to shScramble-MC38 cells (Figure 5h). Quantitation of nodules shows that shGFAT-MC38 cells yielded one or two measurements, just at the level of detection (Figure 5i). Thus, GFAT silencing dramatically inhibits metastasis of this aggressive tumor.

**Figure 6:**
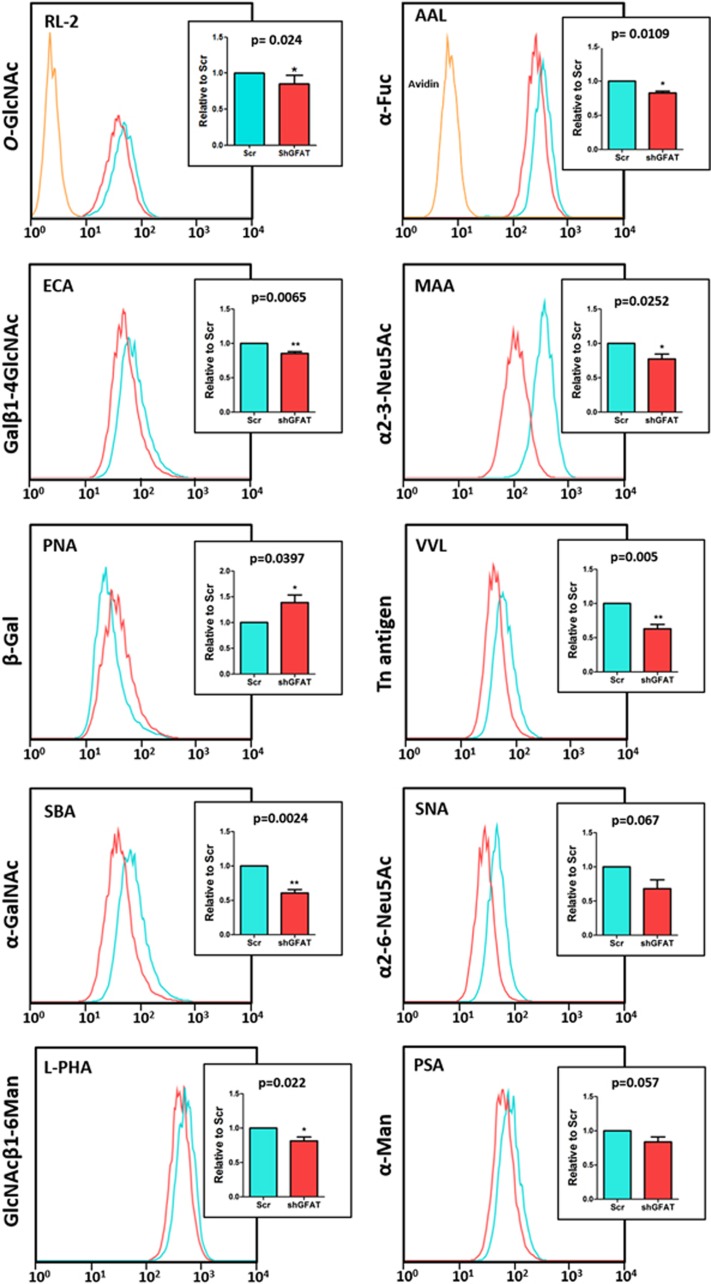
Reduction of aberrant glycosylation in GFAT silenced MC38 cells. Flow cytometry histograms of lectin binding and *O*-GlcNAc immunolabeling. The histograms show representative binding profile of different lectins and *O*-GlcNAc immunolabeling in Scrambled MC38-HG cells (Scr, green) or shGFAT MC38-HG cells (red). Yellow empty histogram refers to cells stained with the FITC-conjugated streptavidin and Alexa Fluor 488-conjgated antibody. Bar graph shows the differences between the fluorescence intensity for each marker. *n*=4 per group; error bars indicate mean±s.e.m. Unpaired *t*-test.

Figure 6 shows that GFAT downregulation significantly decreased expression of triantennary *N*-glycans branched with β1-6GlcNAc as seen by decrease of L-PHA binding (*P*=0.0218). Likewise, decrease of GFAT expression caused a drop in the biosynthesis of Tn antigens (VVL binding). The decrease in SNA binding (*P*=0.0252) with an associated increase of terminal β-Gal*p* unities recognized by PNA (*P*=0.0397) and ECA (*P*=0.0065) upon GFAT downregulation is consistent with the participation of HBP in glycan decoration by α2-6-linked Neu5Ac. It is well established that *O*-GlcNAcylation is highly responsive to HBP.^20^ Here, we analyzed the *O*-GlcNAcylation by flow cytometry using the RL2 antibody after cell membrane permeabilization. As expected, the *O*-GlcNAc levels were significantly decreased in the shGFAT1 cells.

### GFAT is overexpressed in human colon cancers.

**Figure 7:**
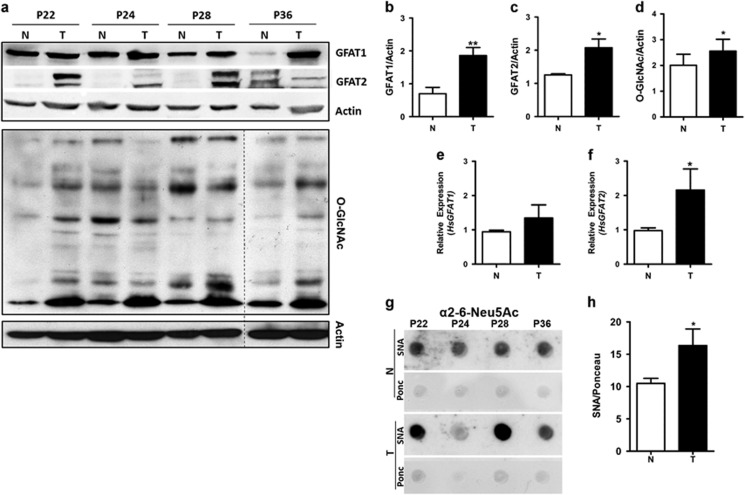
Aberrant levels of GFAT, SNA and *O-*GlcNAc in human colon tissue. (**a**–**d**) Western blot analysis of human colon adenocarcinoma (T) and adjacent normal tissue (N) to GFAT1, GFAT2, *O*-GlcNAc and actin. (**e**, **f**) GFAT1 and GFAT2 mRNA levels in tumor (T) *versus* adjacent normal tissue (N). (**g**, **h**) Dot blot analysis in tumor (T) and adjacent normal tissues (N), measuring the SNA levels. The images are representative from four patients (P22, P24, P28 and P36). All graphs represent means±s.e.m.; one-tailed unpaired *t*-test; total number of seven patients *n*=7; *P<0.05.

Our data suggested an important role for HBP in murine colon cancer and motivated us to investigate whether this pathway is altered in human colon cancer. In this direction, we analyzed GFAT mRNA and protein levels from adenocarcinoma (T) and adjacent normal tissues (N) of colon cancer patients. Our data demonstrated a significant increase of GFAT2 mRNA (Figures 7e and f). Western blot analysis revealed that the protein expression for the GFAT1 (Figures 7a and b), GFAT2 (Figures 7a and c) and *O*-GlcNAcylation (Figures 7a and d) also was remarkably increased in tumor tissue when compared with the adjacent normal tissues. In addition, dot blot analyses revealed a cancer-related increase of sialylation (Figures 7g and h). These data suggest an increase of HBP products in tumor tissues.

## Discussion

It is widely known that aberrant glycosylation is implicated in many cancer types including CRC.^26^ Alterations in cell surface glycosylation directly impact cell growth, survival and promote invasive behavior of tumor cells that, ultimately, lead to metastasis and progression of cancer.^28^ Here we show that increasing extracellular glucose concentration provokes aberrant glycosylation, increased cell proliferation and invasion, presenting an important role in tumor progression. By downregulating GFAT, we demonstrate that HBP is involved in those processes. Hyperglycemic mice show an accelerated development of subcutaneous tumors displaying aberrant glycoconjugates. Furthermore, the experimental metastasis model also presented a more aggressive profile in hyperglycemic mice. Taken together, our results allow us to infer that an increase of glucose levels induces the biosynthesis of aberrant glycoconjugates, and increases tumor progression of murine colon carcinoma cell MC38 through HPB since GFAT downregulation decreases aberrant glycosylation and cell malignancy. In agreement with the experimental data, biopsies from patients with colon carcinoma express more GFAT and aberrant glycans, when compared with adjacent normal tissue. These data suggest an increase of HBP flow in human colon cancer.

Recently, hyperglycemia has been considered a risk factor that links DM to cancer progression.^16, 32, 33^ Hyperglycemia effect has been extensively demonstrated on cell proliferation^15, 34, 35, 36, 37, 38^ and metastasis.^39, 40, 41, 42^ Among those reports an epidemiology study demonstrated that, in cancer patients with DM2 or hyperglycemia, the proportion of tumor recurrence, metastasis, or fatal outcome is higher than that in patients without metabolic disease.^42^ Epidemiological studies also support an antineoplastic role for metformin, an anti-hyperglycemic drug, on cancer risk.^43^ Metformin inhibits cancer cell proliferation *in vitro*^44^ and suppresses colon carcinoma growth *in vivo*.^45, 46^ Furthermore, Metformin treatment can significantly lower the risk of CRC in DM2 patients.^47^ These reports are in agreement with our findings showing that hyperglycemia induces tumor growth, cell invasion homing and metastasis.

Although the underlying mechanisms behind hyperglycemia and cancer association have not been elucidated, inflammation, oxidative stress and immunosuppression are potentially involved in tumor progression in various ways.^48^ Our data showing that incubation of MC38 cells cultured in HG medium was sufficient to induce growing of subcutaneous tumors, homing and metastasis into the lungs of EuG mice rule out the effect of hyperglycemia on host inflammation and immune system. Besides, these results raise the hypothesis that hyperglycemia imprints a memory in MC38 cells (see Discussion below).

Effect of high levels of glucose might be due to an increase of glucose transporters (GLUT),^34, 35, 49^ increasing glucose uptake.^49^ Indeed, tumor cells have a HG uptake through increased expression of GLUT transporters.^50^ Thus, cancer cells might benefit from HG concentration. Once inside the cell, glucose is phosphorylated by hexokinases forming glucose-6-phosphate that can be shunted into the pentose phosphate pathway by glucose-6 phosphate dehydrogenase or undergo enzymatic isomerization, forming fructose-6-phosphate. Even though the majority of glucose enters glycolysis, 2–5% of total glucose within cells enters HBP, generating UDP-GlcNAc, and its derivatives, UDP-GalNAc and CMP-Neu5Ac—donor substrates used in the production of glycoproteins and glycolipids.^19^ Thus, HBP links the altered metabolism with aberrant glycosylation, providing a mechanism of how cancer cells can sense and respond to microenvironment changes. Our group has shown that A549 cells increase glucose uptake during EMT; however, instead of increasing the glycolysis and pentose phosphate pathway, glucose is shunted through the HBP, resulting in a significant increase of UDP-GlcNAc and aberrant glycosylation.^20^ Here we show that MC38-HG cells and tumors from hyperglycemic mice present more UDP-GlcNAc than MC38-LG cell and tumors do from euglycemic mice. Therefore, sustained hyperglycemia would lead to excess UDP-GlcNAc, and its derivatives. Growing evidence demonstrates that alterations in the pool of activated substrates might lead to differential glycosylation.^51^ Also, glycosylation changes are associated with multiple steps of CRC progression.^26^ Our results show that hyperglycemia induces surface glycophenotype alterations both *in vitro* and *in vivo,* favoring malignant cell phenotypes. HG increased the levels of Tn antigen, α2,6-sialylation, α2,3-sialylation, fucosylation, complex β1,6-branched N-linked glycans and intracellular *O*-GlcNAcylation. These results are in accordance with the aberrant glycosylation observed in human colon tumor.^52, 53, 54^ Indeed, α-fucosylation is seen in numerous types of tumors, including colorectal^55, 56, 57, 58, 59^ and it is correlated with tumor metastasis, disease recurrence and poor survival of patients.^60^ However, mutations in GDP mannose-4,6-dehydratase (GMDS) impairing GDP-fucose biosynthesis contribute to the escape of CRC from immune surveillance, indicating that loss of function of fucosylation facilitates cancer progression.^61, 62^ Therefore, enhanced fucosylation has been proposed to be an early event in cancer, whereas glycans are again defucosylated with cancer progression and metastasis.^26, 61, 62, 63, 64^

The expression of Tn antigen (GalNAc-O-Ser/Thr), is associated with earlier stages of CRC, whereas their sialylated counterparts are overexpressed in later stages, also known as metastatic stage.^65, 66^ These results are in agreement with our previous work showing that hyperglycemia provokes aberrant *O*-glycosylation and upregulation of mRNA levels for UDP-GalNAc: polypeptide Nacetylgalactosaminyltransferases (ppGalNAc-T6),^11^ the enzyme that catalyzes the biosynthesis of Tn antigen. Besides, here we observed that hyperglycemia induces an increase of glycoconjugates decorated with α2-6Neu5Ac (SNA labeling). Increased sialic acid on the surface of tumor cells is well described in the literature of several tumor types. Increased expression of α2-6-linked sialic acids on *N*-glycans was associated with cancer progression, occurrence of metastasis, poor prognosis and therapeutic failure in CRC due to decreased cell–cell interactions and increased invasiveness.^67^ There is evidence that all this pro-tumor role of α2-6-linked sialic acids on *N*-glycans is related to maintenance of stemness in CRC cells.^68^ Furthermore, upregulation of ST6Gal1 on colorectal adenocarcinoma cells was shown to increase the α2-6-sialylation of *N*-glycans on β1-integrin adhesion receptors.^69^ Sialylation of these receptors increases their interaction with the cytoskeletal-associated protein talin as well as their binding and haptotactic migration on collagen, thereby leading to enhanced tumor progression. Expression of glycans decorated by α2-3-linked sialic acid (MAA labeling) found here is in accordance with studies showing α2-3-sialylation to be elevated in metastatic colon cancer cell lines.^70^ α2-3-sialylated glycans are major components of cancer-associated sialyl Lewis antigens which play an important role in E-selectin-mediated cancer cell adhesion to vascular endothelial cells during the course of hematogenous metastasis.^71^ Thus, influence of hyperglycemia on expression of sialyl Lewis oligosaccharide by MC38 cells must be verified. Sialylation is often associated with the decrease of outer β-Gal units, thus, reducing labeling by PNA. Given that terminal β-Gal residues are implicated in apoptosis of tumor cells mediated by galectins (a family of carbohydrate-binding proteins with an affinity for β-galactosides, secreted by a variety of cells including the immune system), sialylation on the surface of tumor cells might protect tumor cells from infiltrating immune cells.^72^ Therefore, hypersialylation seen here might favor the evasion of the immune system. It is noteworthy that hyperglycemia increases the expression of complex β1-6-branched N-linked glycan, as seen by increase in L-PHA binding. The β1-6GlcNAc-branched oligosaccharide is a product of Mgat5 activity. Mgat5 activity is limited by UDP-GlcNAc concentration, thus, increased availability of this activated substrate highly increases the β1-6GlcNAc-branched product.^73^ Metabolite availability to the HBP and Golgi increases the number of *N*-glycans and *N*-glycan branching per protein molecule. *N*-glycans are ligands for galectin 1 and 3 at the cell surface, forming lattices that enhance the residence time of receptors as glucose transporters (GLUT4, GLUT2).^74, 75^ *N*-glycosylation indirectly affects metabolism by regulating the surface expression of growth factors receptors, which activate signal transduction pathways that regulate cell growth and metabolism.^73, 75^ Together with previous data,^2, 11, 73, 76^ we might suggest that hyperglycemia influences localization and organization of surface glycoproteins.

UDP-GlcNAc is the donor substrate for OGT, the enzyme responsible for addition of GlcNAc in β-glycosidic linkage to serine or threonine hydroxyls of cytoplasmic and nuclear proteins. Thus, *O*-GlcNAcylation is extremely responsive to increases in glucose uptake.^31, 77^ Onodera *et al.*^38^ showed that *O*-GlcNAcylation of some proteins was observed only in malignant cells, and glucose deprivation or inhibition of HBP or OGT significantly reduced tumor-specific *O*-GlcNAcylation in 3D cultures. Many authors have reported that several cancer types, including breast, bladder, prostate and colon display higher levels of OGT or *O*-GlcNAcylation in grade II or III tumors in comparison to grade I cancers indicating an association with malignancy.^78^ These reports are in agreement with our observation that expression of *O*-GlcNAcylated proteins in patient tumor tissues is higher when compared with the adjacent normal tissues. Although further studies are required, we can suggest that intracellular *O*-GlcNAcylation might control cell surface sialylation, given that it was demonstrated very recently that the activity of UDP-*N*-acetylglucosamine 2-epimerase/*N*-acetylmannosamine kinase (GNE), the key enzyme for the biosynthesis of sialic acids, is modulated by aberrant *O*-GlcNAcylation.^79^

Recently, *O*-GlcNAc has emerged as an epigenetic regulator of gene expression through its influence on higher-order chromatin structure, transcription and modulation of RNA polymerase II.^80, 81^ Thus, it is suggested that *O*-GlcNAcylation might link hyperglycemia to epigenetic.^81, 82^ Indeed, growing evidences have demonstrated that prolonged exposure to HG epigenetically affects gene expression to the level that alterations remain stable in the absence of hyperglycemia, an effect known as Hyperglycemic memory.^83^ Numerous cancerous changes in gene transcription and genome organization have been shown to be highly dependent on epigenetic mechanisms^84^, and epigenetic modulations of oncogenic pathways induced by HG results in prolonged activation of cancer cell proliferation.^85^ Our data suggest that HG prints glycophenotype memories in MC38 cells that remain stable out of the hyperglycemic environment, which are recovered under high blood glucose. Epigenetic regulation of glycosylation is a relatively new emerging concept but with enough studies completed to highlight its importance.^86, 87, 88^ *MGAT5B*, the coding gene for *N*-glycan branching enzyme GnT-IX, is regulated by epigenetic mechanism involving chromatin activation by the OGT–TET3 complex.^89, 90^ In future studies, it would be interesting to investigate the influence of hyperglycemia in histone modifications for the other glycan genes.

To evaluate the importance of GFAT in tumorigenesis of colon cancer, we modulated the flux of substrates through HBP. Apart from using HG, another method for driving increase in HBP influx is to treat cells with GlcNAc, which enters the HBP, bypassing GFAT. Alike observed for HG, GlcNAc increased cell invasiveness. Oppositely, GFAT pharmacological inhibition by DON decreased cell invasion. Data showing that GlcNAc reverts DON inhibition proves that its effect on cell invasion is due to GFAT inhibition. Next, we silenced the rate-limiting enzyme GFAT1. This strategy allowed us to show that GFAT1 controls tumor growth and invasion, and aberrant glycosylation. Corroborating these data, we observed an increase in the levels of GFAT1 and GFAT2, as well as *O*-GlcNAcylation and sialylation, in human colon adenocarcinomas, suggesting an important role of HBP in cancer progression. GFAT overexpression has been observed in prostate cancer biopsies before^91^, and its activity appears to be associated with postprandial hyperglycemia in DM2 patients.^23^ In summary, our data suggest that HBP may be altering the tumor cell biology and accelerating the malignancy process through aberrant glycosylation. Our results suggest the HBP as one mechanism by which hyperglycemia is involved in cancer progression and may point toward a potential pathway amenable for therapeutic intervention.

## Materials and methods

### Cell culture

Mouse colon adenocarcinoma cell line MC38 and the engineered MC38 cells expressing Green fluorescent protein^92^ were kindly donated by Ajit Varki (University of California, San Diego, CA, USA). The human alveolar basal epithelial A549 cells were purchased from American Type Culture Collection (ATCC, USA). All cell lines were maintained in Dulbecco’s modified Eagle medium with high (25 mM) glucose concentration, containing 1% glutamine, 1% pyruvate and 10% fetal bovine serum, 20 mg/ml gentamicin (Sigma-Aldrich, St Louis, MO, USA, 50 mg/ml) at 37 °C in a humidified atmosphere of 5% CO_2_. These cells were switched from high to LG (5 mM) for more than one month before experiments, forming two groups indicated as LG and HG. These conditions are indicated in the figures and legends.

### Population doubling time – (PDT assay)

PDT is the time by which cell population doubles in number. MC38 cells were harvested at 90% confluence and counted. After plating the constant number of cells (10^3^ cells) to a cell culture dish w/2 mm grid (Nalge Nunc International, New York, NY, USA), cells were counted at daily interval for 5 days. Doubling time was calculated based on the log phase of the growth curve acquired by the counting of the cells according to^93^, using the formula:


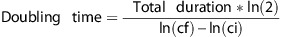


where ‘cf’ is the final cell concentration and ‘ci’ is the initial cell concentration. For total duration, any unit of time can be used and doubling time unit will be the same.

### Cell invasion assay

Transwell membranes (polycarbonic membrane, diameter 6.5 mm, pore size 8 μm—Costar, Cambridge, NY, USA) were coated with matrigel in a total of 100 μl each transwell overnight in a full functioning laminar flow cabinet to let the matrigel solidify (matrigel: 0.375 mg/ml). Afterwards, 2 × 10^5^ MC38-GFP cells were seeded in 200 μl of DMEM-free serum onto the upper chamber, and 900 μl medium with 10% fetal calf serum was added to the lower chamber. After 24 h of incubation, cells adhering to the upper surface of the membrane were removed with a cotton swab. The migrated cells, which adhered to the lower surface, were fixed with 4% paraformaldehyde, and the number of cells attached to the lower surface was counted in the microscopy at × 10 magnification.^94^ Data were obtained from three independent experiments.

### RNA isolation and RT-qPCR

The samples were subjected to phenol–chloroform protocol for total RNA extraction using the TRIzol Reagent (Invitrogen, Carlsbad, CA, USA) according to the manufacturer’s instructions, and RNA concentration was determined using a Nanodrop ND-1000 (Thermo Scientific, Wilmington, NC, USA). The RNA integrity was assessed by native agarose gel electrophoresis. RNA (1 μg) was treated with RNAse-free DNase I (Fermentas International, Burlington, Canada) to eliminate genomic contamination, and used as template for cDNA synthesis using the High-Capacity cDNA Reverse Transcription Kit (Applied Biosystems, Foster City, CA, USA).

Primers for qPCR were designed using the Primer3 software.^95^ qPCR was performed in a StepOne real-time PCR System (Applied Biosystems) using the SYBR Green PCR Master Mix (Applied Biosystems) under the following conditions: one cycle for 10 min at 95 °C, followed by 40 cycles of 15 s at 95 °C and 45 s at 60 °C. qPCR amplification was performed using specific primers for the target genes. All assays were run in triplicate, with HsGAPDH used as endogenous normalization control. Template controls were run in all qPCR experiments to verify whether reaction mixtures were contaminated with exogenous DNA. Reverse-transcriptase controls were also done to confirm that DNaseI treatment had degraded any genomic DNA possibly contaminating RNA samples, and there was no genomic DNA amplification. In this condition, *C*_t_ (cycle threshold) values for all analyzed genes were the same as in no template controls. In the statistical analysis of the qPCR results, the relative expression and ^ΔΔ^*C*_t_ values were calculated from obtained *C*_t_ values, as described elsewhere.^96^ The ^ΔΔ^*C*_t_ mean values obtained from the experiments were submitted to Grubb's test to detect outliers^97^, and the comparison among different conditions was made using Student’s *t*-test and differences were considered significant at *P*<0.05. The relative expression values (2^−ΔΔ^C_t_) were used only for graph construction. Statistical analysis was performed using the SAS software version 9.1.3 (SAS Institute, Cary, NC, USA).

### Lentivirus construction and transduction of MC38 cells

Lentivirus particles were produced with pGFP-C-shScrambled or shGFPT1 plasmid (Origene, TL511601) and lentivirus packaging plasmids (Invitrogen) according to manufacturers' instructions.

### Flow cytometry

The cell lectin binding was performed as described.^11^ For *O-*GlcNAc immunolabeling, 1 × 10^6^ MC38 cells were fixed with 3.7% formaldehyde (Merck, Darmstadt, FRG) in phosphate-buffered saline (PBS) for 15 min at 4 °C. Cells were pelleted by centrifugation at 300 g for 5 min, permeabilized for 15 min with 0.2% v/v Tween 20 (Sigma) in PBS at 37 °C, and washed once with PBS. Permeabilized cells were incubated overnight at 4 °C with a 1:100 dilution of anti-*O*-GlcNAc antibody (RL2; SC59624, Santa Cruz Biotechnology, Dallas, TX, USA) in PBS (100 μl final volume). Cells were washed twice and followed by incubation at 4 °C for 2 h in 100 μl of a 1:2500 dilution of Alexa Fluor 488-conjgated antibody (A-11001, Invitrogen) in PBS. Final cell pellets were washed twice and suspended in PBS for flow cytometric acquisition of 10 000 events with a FACSCalibur flow cytometer (BD Biosciences, San Jose, CA, USA).^98^ The fluorescence intensity data were analyzed with FlowJo X.

### Animals

Experiments were performed on adult male C57BL/6 (3–4 months old) in accordance with the National Institutes of Health Guide for the Care and Use of Laboratory Animals (NIH Publication no. 80–23), and were approved by the Committee for the Use of Experimental Animals of our institution. Animals were selected using a simple random method. Hyperglycemic mice were induced with a single intra-peritoneal (i.p.) injection of 150 mg/kg STZ (Sigma-Aldrich) diluted with 0.1 M sodium citrate buffer (pH 4.3) prior to use. Control mice received 0.1 M sodium citrate buffer (pH 4.3) in a single i.p. injection as vehicle. Blood was collected weekly from tail veins, and blood glucose levels were measured using a validated one-touch basic glucose measurement system (Accu-chek Active Test meter, Roche) and body weight was also measured weekly from baseline (Supplementary Figures 1A,B). Seven days after STZ treatment, the mice with blood glucose concentrations >300 mg/dl received cell infusion. No blinding investigator was used in the animals’ experiments.

### Tumor growth assay

Subcutaneous tumor was induced by implantation of 1 × 10^5^ MC38 cells on the right flank of C57BL/6. Animals were separated into the following six experimental groups: (1) mice injected with cells cultured in LG condition (MC38-LG); (2) mice injected with cells cultured in HG condition (MC38-HG); (3) hyperglycemic mice injected with cells cultured in HG condition (MC38-HG-HyG); (4) mice injected with cells cultured in HG condition and pre-treated with DON (MC38-HG-DON); (5) mice injected with cells cultured in HG condition and silenced to GFAT1 (MC38-HG-shGAT); and (6) mice injected with cells cultured in HG condition and scrambled to GFAT1 (MC38-HG-shGAT). The tumor was measured twice a week for 28 days, and tumor area was calculated. Growth curves for tumors were plotted as the mean area±s.e.m. of tumors of mice from each group. No statistical method was used to predict sample size and size of the sample varied from 9 to 18 per group, specified in each figure’s legends.

### Experimental lung metastasis

As an animal model of lung metastasis, 2 × 10^5^ MC38 cells were inoculated in 100 μl of DMEM via tail vein of the mice. After 22 days, mice were anaesthetized with an i.p injection of Ketamine/xylazine 100 mg/kg and 10 mg/kg body weight, respectively, and transcardially perfused with PBS. A picture of dissected lungs was taken and a GFP fluorescence image was obtained in IVIS Lumina System. Further, lungs were processed for quantification of metastasis by detection of GFP fluorescence. Perfused lungs were homogenized in 2 ml of a hypotonic buffer (20 mM Tris-Cl, pH 7.0) by using a potter. Triton X-100 was added to a final concentration of 0,5 %. After 30 min on ice, the insoluble debris was spun down (10 000 g for 10 min), 10 μl of each supernatant was diluted with Tris-Cl buffer (20 mM Tris-Cl, pH 7.0) to a final volume of 100 μl, and transferred to a quartz cubet. Fluorescence was read on a Varian Cary Eclipse (Varian) at Ex 485 and Em 508. Background levels for subtraction were determined on lungs from mice, which had not been injected with GFP-labeled cells.^92^

### Tissue lysates and western blot

Total lysates were obtained homogenizing tissue samples in a potter with lysis buffer 1% Triton X-100, 0.5% sodium deoxycolate, 0.2% SDS, 150 mM NaCl, 2 mM EDTA, 10 mM HEPES (pH 7.4), 20 mM NaF, 1 mM orthovanadate and a protease inhibitor cocktail, for 30 min at 4 ^o^C. After centrifugation at 10 000 g for 10 min at 4 °C, the supernatant was removed and stored at −80 °C. For immunoblotting equal amounts of protein (30 μg/lane) from tissue lysates were separated on 7.5% SDS–PAGE and transferred onto nitrocellulose sheets. The membranes were blocked and incubated overnight with primary antibodies GFAT1 and GFAT2 (SC134894 and SC134710, respectively, Santa Cruz Biotechnology), CTD-110.6 for *O*-GlcNAc (SC-59623, Santa Cruz Biotechnology), β-actin (A5316, Sigma Chemical) and vinculin (V4505, Sigma Chemical). After washing, the membranes were incubated for 1 h with peroxidase-conjugated secondary antibodies, developed using ECL (GE Healthcare, Pittsburgh, PA, USA) and exposed to Image Quant LAS 4000 (GE Healthcare). ImageJ software was used for densitometry analysis of immunoblots, and measurements were normalized against β-actin or vinculin loading controls.

### Lectin binding in tissue sections

Animals were deeply anesthetized with an intraperitoneal injection of Ketamine/xylazine 100 mg/kg and 10 mg/kg body weight, respectively, and were transcardially perfused with 4% paraformaldehyde in 0.1 M PBS, pH 7.4. Tissue was cryoprotected in 30% sucrose in 0.1 M TPO_4_ for at least 1 week, then embedded in O.T.C compound (Meio Tissue-Tek, Sakura) and cryosectioned into 20-μm sections. Lectins and their nominal sugar specificities are listed in Supplementary Table 1. Staining was performed at 4 °C overnight to 100 μg/ml. Negative controls consisted of streptavidin alone and biotinylated lectins without fluorescent streptavidin. Fluorescent samples were analyzed under a confocal microscope (Zeiss LSM 510 Meta).

### Dot blot

For dot blot analysis, 10 μg of total cell lysates were spotted and immobilized in nitrocellulose membrane. The membrane was blocked with 3% of bovine serum albumin (BSA) and incubated overnight with biotinilated *Sambucus nigra*Lectin (SNA, Vector Laboratories), α2-6-Neu5Ac labeling. The blots were then washed, incubated with Extravidin-peroxidase (Sigma), developed using ECL (GE Healthcare) and exposed to Image Quant LAS 4000 (GE Healthcare). ImageJ software was used for densitometric analysis, and measurements were normalized against Ponceau staining.

### Magnetic resonance imaging

The initial development of subcutaneous tumor was analyzed *in vivo* by magnetic resonance imaging measurements 5 days after MC38 cells injection. The animals were anesthetized with Isoforine and received an intravenous injection (0.5 ml/kg weight body) with the contrast agent gadolinium (Dotarem; 0.5 mmol/ml). Images were acquired with a 7T magnetic resonance scanner (Varian MRI System 7T/210 ASR Horizontal Bore Magnet, Agilent Technologies, Santa Clara, CA, USA). Three T1-weighted spin echo sequences were acquired (axial with TR/TE=420 ms/15 ms, matrix=128 × 128, FOV=80 mm/50 mm, 20 slices, no gap, thickness=1 mm, NEX=10; coronal with TR/TE=750 ms/15 ms, matrix=128 × 128, FOV=35 mm/35 mm, 35 slices, no gap, thickness=1 mm, NEX=10; sagittal with TR/TE=525 ms/15 ms, matrix=128 × 128, FOV=80 mm/40 mm, 25 slices, no gap, thickness=1 mm, NEX=10) and a T2-wieghted fast spin echo sequence (TR/ TE=2520 ms/15 ms, matrix=128 × 128, FOV=80 mm/50 mm, 20 slices, no gap, thickness=1 mm, NEX=10)

### UDP-hexosamines detection

UDP-hexosamines produced by MC38 cells *in vitro* were determined by HPLC using a Hypercarb PGC column (3.0 mm × 150 mm, Thermo Scientific) as described before^20^.

The presence of UDP-hexosamines on-tissue samples was revealed by high-resolution matrix-assisted laser desorption/ionization Fourier-transform ion cyclotron resonance mass spectrometry imaging (MALDI-FT-ICR MSI). Subcutaneous tumor from euglycemic and hyperglycemic mice were frozen in liquid nitrogen and stored at −80 °C. Frozen (12 μm) sections were mounted onto indium-tin-oxide (ITO)-coated glass slides (Bruker Daltonik, Bremen, Germany) pretreated with 1:1 poly-L-lysine (Sigma Aldrich, Munich, Germany) and 0.1% Nonidet P-40 (Sigma) for MSI analysis. Adjacent sections were mounted on glass slides for hematoxylin and eosin (H&E) staining. All sections were stored at −80 °C until analysis. Prior to matrix application, tumor sections were dehydrated in a vacuum for 1 h at room temperature. Samples were coated in 10 mg/ml 9-aminoacridine matrix in 70% methanol using an ImagePrep spray roboter (Bruker Daltonics, Billerica, MA, USA). MSI was performed in negative ion mode on a Bruker Solarix 7 T FT-ICR MS (Bruker), over a mass range of *m/z* 150–1000 and 50 μm lateral resolution. All measurements included a non-tissue measurement region as a background control. Acquired data were analyzed using SCILS Lab (Bruker Daltonics) and normalized against the root mean square of all data points. MS/MS was conducted on On-tissue samples using electron capture dissociation in the collision cell.

### Patient samples

Colon adenocarcinoma and adjacent normal tissues derived from biopsy of seven patients (four males and three females) were obtained from the Instituto Nacional de Câncer (INCA), Rio de Janeiro, Brazil, which banked samples following patient consent. All samples were from surgical resections and were evaluated by a board-certified pathologist. In all cases, control specimens were collected from the accompanying normal mucosa, distant approximately 10 cm from the carcinoma. The cancer tissue and the normal epithelial layer were carefully isolated from the resected colon with scissors and forceps. All tumor samples used were determined to be >80% tumor by evaluation of H&E-stained sections. H&E-stained sections of each normal sample were evaluated and determined to lack any precancerous lesions. Clinic pathologic features are listed in Supplementary Table 2. Samples for immunoblotting were frozen at −80 °C and those used for qRT-PCR were immediately immersed in RNA later stabilization solution (Ambion–Life Technologies, Carlsbad, CA, USA) and stored at 4 °C overnight, then samples were moved to −80 °C for long-term storage. This study was carried out with approval of the Brazilian National Cancer Institute’s Ethic Committee (Registration number: 84/04).

### Statistical analysis

All data were analyzed in Prism (Graphpad). One/two-tail unpaired *t*-test or one/two-way ANOVA (Dunnett, Tukey’s/Bonferroni post test) was used when comparing two groups or more than two groups, respectively. Error bars represent mean±s.e.m. when not specified.

### Study approval

All experiments involving mice were approved by the Institutional Animal Care and Use Committee and followed all state and federal rules and regulations. All human samples used in experiments were deidentified and obtained from the Instituto Nacional do Cancer, which banked samples following informed consent.
