# Supplementary material for: Hyperglycemia exacerbates colon cancer malignancy through hexosamine biosynthetic pathway
Source: Oncogenesis. 2017 Mar 20;6(3):e306–. doi: 10.1038/oncsis.2017.2 (PMC5533945; doi:10.1038/oncsis.2017.2)
Supplement: Supplementary Information [file oncsis20172x1.docx]

**SUPPLEMENTAL INFORMATION**

**Hyperglycemia exacerbates colon cancer malignancy through hexosamine biosynthetic pathway**

Andréia Vasconcelos-dos-Santos^1^, Hector Loponte^1^, Natalia Rodrigues Mantuano^1^, Isadora de Araujo Oliveira^1^, Iron Francisco de Paula^2^, Leonardo K. Teixeira^3^, Julio Cesar Madureira de-Freitas-Junior^2^, Katia Calp Gondim^2^, Norton Heise^1^, Ronaldo Mohana Borges^1^, José Andrés Morgado-Díaz^2^, Wagner B. Dias^1^and Adriane R. Todeschini^1^*

*^1^Instituto de Biofísica Carlos Chagas Filho, ^2^Instituto de Bioquímica Médica Leopoldo de Meis, Universidade Federal do Rio de Janeiro, ^3^Programa de Biologia Celular, Instituto Nacional de Câncer (INCA), Rio de Janeiro, RJ, Brazil*.


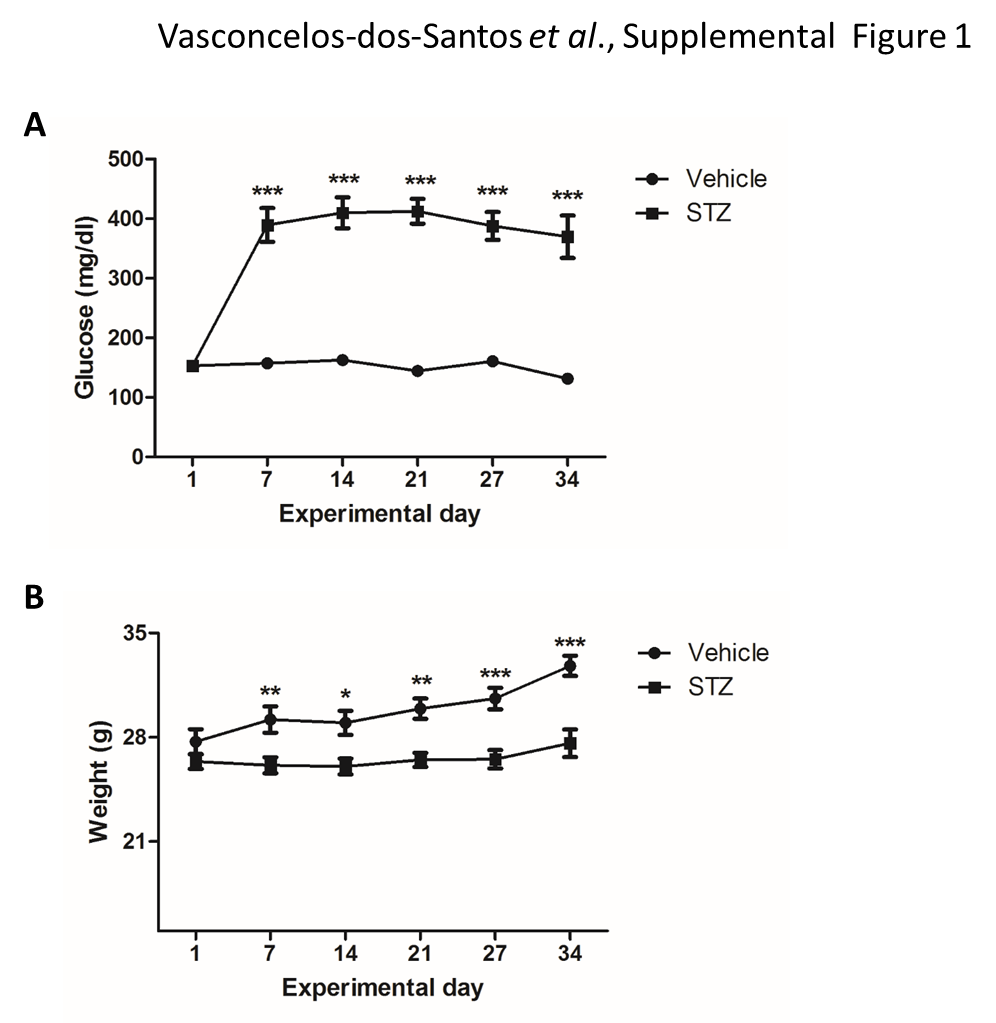
**Supplemental Figure 1**

**Figure S1. Glucose levels of STZ treated animals**.

(A) Blood glucose levels.

(B) Body weight for the Euglycemic (Vehicle) and hyperglycemic (STZ-treated) group. Data are shown as mean ± SEM. *p < 0.05, **p<0,001, ***P < 0.001. (n=25-30).

**
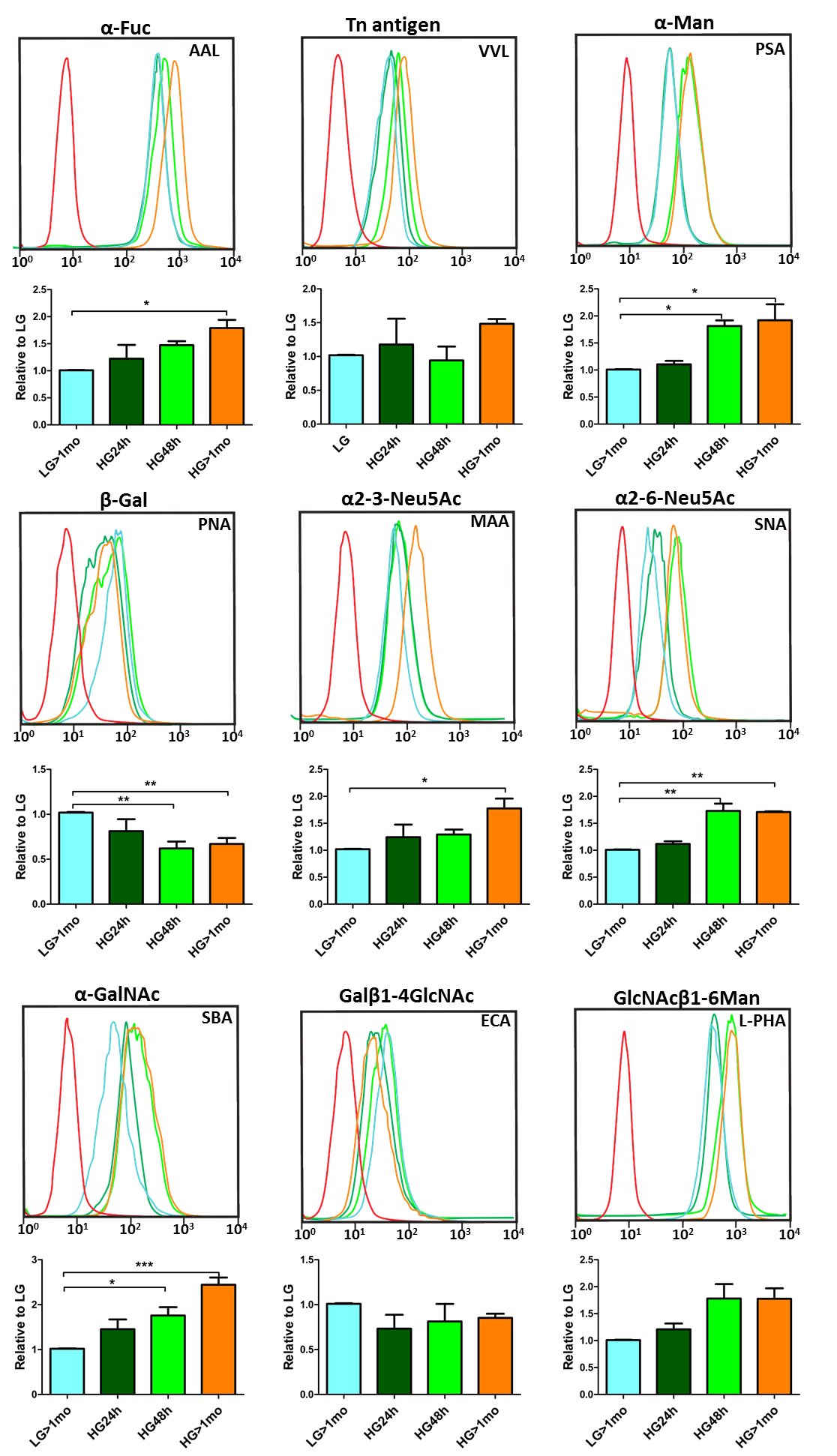
Supplemental Figure 2**

**Figure S2. Time dependent effect of hyperglycemia on MC38 cells glycosylation.**

Flow cytometry histograms show representative binding profile of different lectins in MC38 cells cultured in different times and concentration of glucose: Low glucose for more than 1 month (LG>1mo, blue), high glucose for 24h (HG24h, green), high glucose for 48h (HG48h, light green) and high glucose for more than 1 month (HG>1mo, orange). Red empty histograms refer to cells stained with the FITC-conjugated streptavidin and bar graph shows the differences between the fluorescence intensity for each lectin relative to LG condition. The results represent four experimental replicates. (n=4); Unpaired t test.

**Supplemental Figure 3**

**
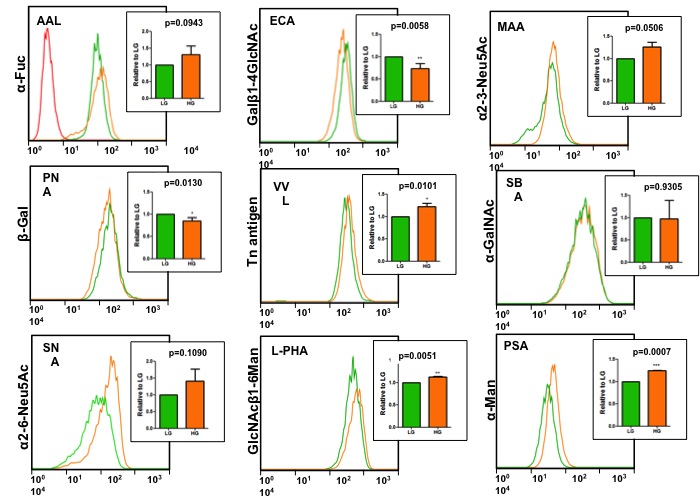
**

**Figure S3. Hyperglycemia induces aberrant glycosylation in 4T1 cells.**

Flow cytometry histograms show representative binding profile of different lectins in 4T1 cells cultured in low (LG, green) or high glucose (LG, purple) concentration (both was cultured for more than 1 month in each conditions). Red empty histogram refers to cells stained with the FITC-conjugated streptavidin and bar graph shows the differences between the fluorescence intensity for each lectin relative to LG condition. The results represent four experimental replicates. (n=3).

**Supplemental Figure 4**

**
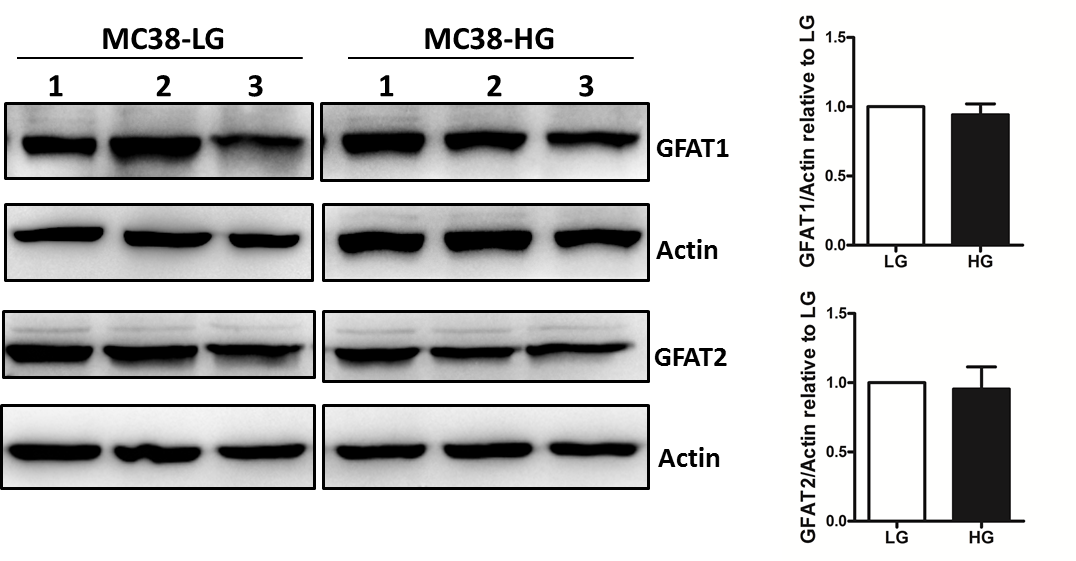
**

**Figure S4. GFAT 1 and 2 expressions pattern in MC38-LG versus MC38-HG**.

(A-B) Western blot analysis of GFAT 1 and 2 expressions in MC38-LG compared to MC38-HG cells. Blots shown are representative results in 3 different samples. Data are shown as mean ± SEM. (n=14).

**Supplemental Figure 5**





**Figure S5. Lung homing of MC38-GFP cells cultured in HG and treated or not with DON**. MC38-HG GFP^+^ cells were injected into lateral tail vein of Euglycemic mice and analyzed three days after infusion. Data are shown as mean ± SEM (n=10).

**Supplemental Table 1.** Specificity of different Lectins

| ***Lectin*** | ***Abbreviation*** | ***Specificity*** |
| --- | --- | --- |
| *Maackia amurensis* agglutinin | MAA | α2-3-linked Neu5Ac |
| *Sambucus nigra* agglutinin | SNA | α2-6-linked NeuAc |
| *Aleuria aurantia* lectin | AAL | α-Fuc |
| *Phaseolus vulgaris* agglutinin | PHA-L | Galβ1-4GlcNAcβ1-6Man |
| *Erythrina cristagalli* agglutinin | ECA | Galβ1-4GalNAc |
| Peanut agglutinin | PNA | Terminal β-Gal*p* |
| *Pisum sativum* agglutinin | PSA | Mannose |
| Soybean agglutinin | SBA | α-GalNAc |
| *Vicia villosa* lectin | VVL | Tn antigen |

**Supplemental Table 2**: Clinic pathologic features

| SAMPLE SIZE | 7 |
| --- | --- |
| **Gender** |  |
| M/F | 4/3 |
| **Age (years)^1^** | 62 ± 8 |
| **Location** |  |
| Ascending colon | 1 |
| Transverse colon | 2 |
| Sigmoid | 1 |
| Rectosigmoid | 3 |
| **Histology^2^** |  |
| Well | 0 |
| Moderately | 7 |
| Poorly | 0 |
| Mucinous | 0 |
| **TNM stage** |  |
| 0 | 0 |
| I | 0 |
| II | 3 |
| III | 4 |
| IV | 0 |
|  |  |

^1^Value are mean ± Standard deviation. ^2^Well, well-differentiated adenocarcinoma;

moderately, moderately differentiated adenocarcinoma; poorly, poorly or

undifferentiated adenocarcinoma; mucinous, mucinous adenocarcinoma.
